# Melioidosis in Hong Kong

**DOI:** 10.3390/tropicalmed3030091

**Published:** 2018-08-25

**Authors:** Grace Lui, Anthony Tam, Eugene Y. K. Tso, Alan K. L. Wu, Jonpaul Zee, Kin Wing Choi, Wilson Lam, Man Chun Chan, Wan Man Ting, Ivan F. N. Hung

**Affiliations:** 1 Department of Medicine and Therapeutics, Faculty of Medicine, Stanley Ho Centre for Emerging Infectious Diseases, The Chinese University of Hong Kong, Hong Kong, China; 2 Department of Medicine, Queen Mary Hospital, Hong Kong, China; antamwf@connect.hku.hk; 3 Department of Medicine and Geriatrics, United Christian Hospital, Hong Kong, China; tsoyke@ha.org.hk; 4 Department of Clinical Pathology, Pamela Youde Nethersole Eastern Hospital, Hong Kong, China; alanklwu@gmail.com; 5 Department of Clinical Pathology, Tuen Mun Hospital, Hong Kong, China; jonpaulzee@gmail.com; 6 Department of Medicine, Alice Ho Miu Ling Nethersole Hospital, Hong Kong, China; choikw1@ha.org.hk; 7 Department of Medicine, Queen Elizabeth Hospital, Hong Kong, China; lwzz04@ha.org.hk (W.L.); twm608@ha.org.hk (W.M.T.); 8 Department of Medicine and Geriatrics, Princess Margaret Hospital, Hong Kong, China; cmc061@ha.org.hk; 9 Carol Yu Centre for Infection, Department of Medicine, Li Ka Shing Faculty of Medicine, The University of Hong Kong, Hong Kong, China; 10 Department of Clinical Microbiology and Infection Control, The University of Hong Kong-Shenzhen Hospital, Shenzhen 518172, China

**Keywords:** melioidosis, *Burkholderia pseudomallei*, Hong Kong

## Abstract

Melioidosis, although endemic in many parts of Southeast Asia, has not been systematically studied in Hong Kong, which is a predominantly urban area located in the subtropics. This review describes the early outbreaks of melioidosis in captive animals in Hong Kong in the 1970s, as well as the early reports of human clinical cases in the 1980s. A review of all hospitalized human cases of culture-confirmed melioidosis in the last twenty years showed an increasing trend in the incidence of the disease, with significant mortality observed. The lack of awareness of this disease among local physicians, the delay in laboratory diagnosis and the lack of epidemiological surveillance are among the greatest challenges of managing melioidosis in the territory.

## 1. Introduction

Melioidosis is an emerging infectious disease caused by *Burkholderia pseudomallei* that causes significant morbidity and mortality [1]. Melioidosis was first described in Southeast Asia in the early 1910s [2]. Since then, melioidosis has been well recognized to be highly endemic in northern Australia and many parts of Asia, with the highest prevalence observed in Southeast and South Asia [3,4]. It is being increasingly reported in previously non-endemic regions [4].

Melioidosis is endemic in regions located at tropical latitudes between 20° N and 20° S [1]. However, there is an increasing bulk of evidence of this disease in territories with no previous documentation of melioidosis, as well as an expansion of the geographical areas with a significant burden of the disease outside the tropics, including Southern China [5,6] and Taiwan [7]. Hong Kong is also considered to be situated in an endemic region with high environmental suitability for *B. pseudomallei* [3,4].

Despite its high fatality rate, melioidosis is often a neglected disease. It has been estimated that the disease was regularly under-reported in many countries [4]. *B. pseudomallei* is a natural saprophyte, thus human cases of melioidosis were frequently associated with exposure to soil and contaminated waters in rural areas [8]. Epidemiological studies from urbanized cities like Hong Kong were therefore rarely performed. Melioidosis is currently not a statutorily notifiable disease in Hong Kong, and this disease has never been systematically studied in Hong Kong. Therefore, the local burden of illness is currently largely unknown.

This review aims to provide an overview of melioidosis in Hong Kong. In the first part, the history of melioidosis in humans and animals and evidence of *B. pseudomallei* in the environment, as reported in the literature, is reviewed. In the second part, a review of all clinical cases of culture-confirmed melioidosis managed in public hospitals in Hong Kong in the past 20 years is presented. This is followed by results of a survey performed among local infectious diseases physicians and microbiologists on the local practice of diagnosis and management of melioidosis. Lastly, challenges in the control and clinical management of this disease are discussed.

## 2. History of Melioidosis in Hong Kong: Presence in Animals, Humans and the Environment

The earliest record of *B. pseudomallei* in Hong Kong came from isolates obtained from bottlenose dolphins and a harbor seal kept in an oceanarium in 1975 and 1976 [9]. This outbreak at the oceanarium caused 24 dolphins to succumb to this illness [10]. Subsequently, *B. pseudomallei* was isolated from other dolphins and numerous other captive sea mammals, including sea lions and pilot whales, as well as birds in a nearby aviary, including zebra doves and scarlet macaws, from 1978 to 2000 [9,11]. 

A clinical review was performed to evaluate all the melioidosis cases in animals kept at the oceanarium in the period from 1974 to 2003. A total of 49, 31, 16 and 5 cases of melioidosis were recorded in cetaceans, pinnipeds, birds and terrestrial mammals during this period, respectively. All but four succumbed to the disease. Melioidosis caused 55% and 50% of all known causes of deaths in cetaceans and pinnipeds, respectively, during this period [12].

An experimental acellular *B. pseudomallei* vaccine that was tested to be safe and effective in hamsters was given to false killer whales and bottlenose dolphins in the oceanarium from 1986 to 1987. The vaccine produced a high level of specific antibodies in these cetaceans, and was able to reduce mortality in the cetaceans from 45% in 1983 to less than 1% in 1988 [13].

The first human case of melioidosis in Hong Kong was reported in 1983 [14], followed shortly by another five cases reported in 1984 [10]. All these patients were immunocompromised, with either diabetes or had been receiving immunosuppressive therapy for autoimmune diseases. All patients had bacteremia with or without pulmonary involvement. None of them had reported travelling to other endemic areas. Mortality was high, and the majority of them did not receive adequate anti-microbial therapy.

The first serological study was published in 1984, in which 22 elderly patients admitted to a general medical unit of a teaching hospital were screened for hemagglutinating antibodies against lipopolysaccharide antigens of *B. pseudomallei*. 23% of patients in this small cohort had evidence of possible subclinical infection [10,15]. This was followed by another serological survey published in 1987 involving 275 patients with underlying tuberculosis or chronic pulmonary diseases. This study showed that 14% of subjects had serological evidence of exposure to *B. pseudomallei*. Among those with positive antibodies against *B. pseudomallei*, 77% of subjects had not traveled to other endemic areas [15]. This finding, together with the first few human cases described above, produced the earliest evidence that melioidosis was also endemic in Hong Kong, and that human exposure to the bacterium was not at all rare.

Subsequent to those early case reports, eight more human cases of culture-confirmed melioidosis were published from 1987 to 2015 (Table 1). Most of these patients had underlying immunocompromised conditions, including diabetes and hematological malignancies with prolonged neutropenia. One 40-year-old man, with concomitant miliary tuberculosis, was found to have X-linked chronic granulomatous disease. Most patients had bacteremia, pulmonary infection or deep organ abscess. One 82-year-old man developed a mycotic aneurysm in the aortic arch. Such clinical presentation had been reported rarely elsewhere [16]. All of these subsequently reported cases received either intravenous ceftazidime or carbapenem as induction therapy. Three out of these eight patients died due to melioidosis.

There was also a case series published in 2010, describing five patients with autoantibodies against interferon-γ with positive IgG or IgM antibodies against *B. pseudomallei*. All these patients had concomitant non-tuberculous mycobacteriosis, penicilliosis and/or non-typhoidal salmonellosis. None of these patients had a positive culture of *B. pseudomallei* [17].

As in neighboring regions located at similar latitudes, where *B. pseudomallei* had been isolated from the soil [7,18], the bacterium was also isolated from the environment in soil samples in Hong Kong as early as the 1970s [9]. These early reports showed that *B. pseudomallei* was isolated in soil samples taken from sea sludge, farms and a children’s playground [10]. In 2000, multiple soil and water samples from an aviary were also found to harbor the bacterium [9]. More recently, among 1400 soil samples collected from the oceanarium in Hong Kong, over a 15-month period, 6.8% of the samples were positive for *B. pseudomallei* by PCR [19].

An epidemiological study, using multilocus sequence typing, on isolates obtained from captive animals and the environment from 1975 to 2000 in Hong Kong, showed that there were two major clusters involving three serotypes. All of these serotypes had been identified in human cases of melioidosis from Hong Kong [9]. The seasonality of melioidosis cases among the captive animals also correlated with the timing of detection of *B. pseudomallei* in soil samples [14]. Such epidemiological data suggested that humans, as in the case of animals, acquired the same strains of *B. pseudomallei* from direct exposure to soil and water [9].

## 3. Review of Culture-Confirmed Melioidosis Cases in Humans in the Past 20 Years

To determine the burden of illness caused by melioidosis in Hong Kong, a retrospective review of all patients admitted to all public hospitals in Hong Kong with *B. pseudomallei* isolated from one or more clinical specimens during the period from 1998 to 2017, was conducted. In Hong Kong, public hospitals under the Hospital Authority of Hong Kong provide more than 80% of in-patient services to the population in the territory [20]. All patients with one or more clinical specimens with *B. pseudomallei* isolated were identified from the Clinical Data Analysis and Reporting System (CDARS), which is a central computerized database of the Hospital Authority, including patients’ demographic characteristics, diagnoses, laboratory data and drug treatment.

**Figure 1:**
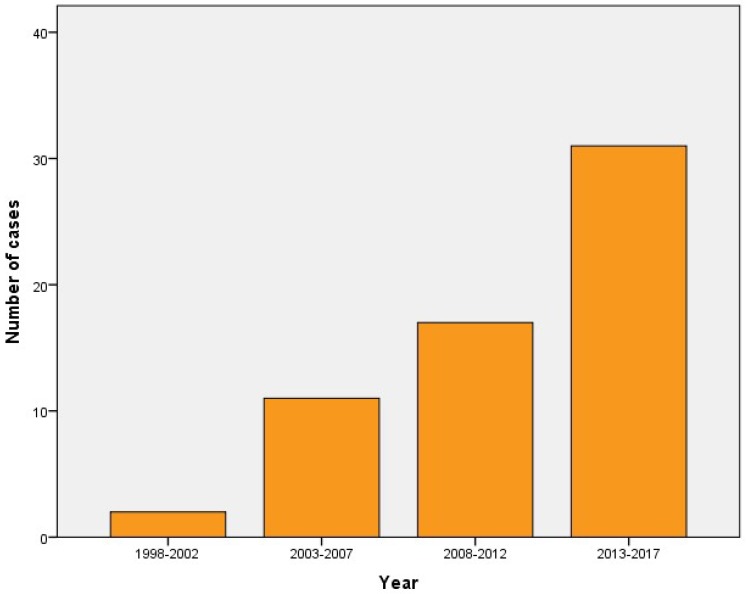
The number of culture-confirmed human cases of melioidosis over a 20-year period.

A total of 61 culture-confirmed cases of melioidosis was identified during this 20-year period. A progressive increase in the number of cases was observed over this period (Figure 1). 72.1% of the subjects were male, and the median age was 67 (range 8–100, interquartile range 58–83). Only three pediatric cases, with an age between 8 and 17 years, were identified. 60.7% of them had bacteremia, 42.6% had a pulmonary infection, 23.0% had deep organ abscesses (most common being prostate, kidneys, spleen and liver), 11.5% had skin and soft tissue infection and 6.5% had bone and joint infection. A relapse of melioidosis was observed in two patients within 12 months of the initial episode. The overall case fatality rate was 31.1%. Mortality was associated with the presence of bacteremia only (odds ratio 5.14, 95% confidence interval 1.26–21.07, *p* = 0.023).

This series of human cases together with earlier case reports published in the 1980s strongly suggested that melioidosis is endemic in Hong Kong. The saprophytic nature of *B. pseudomallei* has been well recognized since the 1950s [1]. As such, occupational and recreational exposure to soil and contaminated water surfaces, e.g., those working in farms and rice paddies, and those with ingestion or inhalation of food and water contaminated with soil, were consistently described as risk factors for acquiring melioidosis [1,28]. Therefore, not surprisingly, large cohorts of human cases of melioidosis were most commonly reported from rural parts of countries and regions, where primary industry is common [29,30]. Lower incidence of melioidosis was observed in higher-income countries possibly due to lower exposure rates [4]. However, our review and other studies of melioidosis performed in urban cities [31] serve as a good reminder that melioidosis, though less prevalent, should not be neglected in these cities, due to the high mortality and rising incidence. In urbanized areas, working in construction sites, military forces and recreational exposure to soil and contaminated waters have been described as risk factors for acquiring the infection [1,32,33].

We observed a rising trend of culture-confirmed cases over the last 20 years in Hong Kong. An increase in the incidence of human cases of melioidosis has also been reported in Asia in the past decade [4]. For example, Hainan witnessed an increasing trend of annual incidence of melioidosis, with most cases residing in Sanya and Haikou cities [6]. There are several postulations accounting for the rising trend observed in Hong Kong. Improved case detection with better microbiological diagnostic tools is a possibility. Weather changes, through their impacts on environmental contamination, have been shown to cause clustering of human cases of melioidosis [34]. In Hong Kong, the detection rate of *B. pseudomallei* from soil was shown to have significant positive correlation with ambient temperature and relative humidity [19]. Therefore, the significant warming trend and more frequent extreme precipitation events since the 20th century, as reported by the Hong Kong Observatory, could possibly increase human exposure to the bacterium from the environment. The expanding scope of international traveling was proposed to be a source of the importation of cases and the emergence of melioidosis in developed countries as well [4]. Human cases of melioidosis from Hong Kong and other Southeast Asian countries have been shown to share the same serotype [9], supporting the possibility that the disease could be acquired during travel. Lastly, our patients were older than those in other cohorts [29,30], and melioidosis is well recognized to recrudesce after a prolonged period of time [35]. Most of the patients described in the published case reports from Hong Kong had a wide spectrum of underlying immunocompromised conditions (Table 1). Therefore, aging and the increase in immunocompromised diseases and therapies could have contributed to the increase in the incidence of melioidosis by reactivation of the disease after acquisition earlier in life [21,24].

Distinct from the experience in some other Asian countries [36,37], pediatric infections were rare in our cohort. On the other hand, the spectrum of disease involvement observed in our cohort was similar to those described in other studies [35], with bacteremia, pulmonary infection, deep organ abscesses and genitourinary infection being the most common. Bacteremia was present in 61% of cases, and was the major predictor for mortality. The recurrence rate in our cohort was similar to that described previously [35]. Although standard antimicrobial therapy was given to the majority of patients, case fatality rate was still high at 31%. The case fatality rates observed in other developed countries were 14–18% [30,31]. The higher case fatality rate in our cohort could partly be due to the older age of our patients, as compared to most other studies [6,31,38].

## 4. Current Practice of Management of Melioidosis in Hong Kong

An online survey was performed among local infectious disease physicians and microbiologists from May to July 2018 to evaluate the contemporary local practice in diagnosis and management of melioidosis. The survey was sent to 44 infectious disease physicians and microbiologists, of whom 28 (64%) responded to the survey. They had a median of eight years (interquartile range 3–13) of experience as a specialist. The majority of the respondents (57.1%) had managed 1–2 culture-confirmed cases of melioidosis in the past, while 32.2% had seen three or more cases, and 10.7% had never seen a case.

For laboratory diagnosis, 67.9% of the respondents had access to matrix-assisted laser desorption/ionization time-of-flight mass spectrometry (MALDI-TOF MS) as one of the usual methods of identification of *B. pseudomallei*, while 46.4% and 42.9% regarded biochemical and phenotypic testing and Vitek as the usual methods of identification respectively. 25.0% regarded 16S rRNA sequencing as one of the usual methods of identification. The majority (71.4%) did not find serology to be useful in diagnosing melioidosis, mainly due to the perceived low specificity of the assay. 19.2% of the respondents, however, did believe serology would increase case detection, especially in patients who had negative cultures after antibiotics treatment, and those with compatible epidemiological exposure.

All respondents would prescribe either ceftazidime or meropenem for at least two weeks for the initial treatment of melioidosis. 82.1% of them would choose trimethoprim-sulfamethoxazole, and the remainder amoxicillin-clavulanate, for at least three to six months, as an eradication therapy.

The majority of respondents regarded the lack of awareness among clinicians as the greatest challenge in the management of melioidosis in Hong Kong. The relatively small number of cases in Hong Kong limited the experience and expertise for this disease entity. The low level of clinical suspicion would cause a delay in diagnosis. Respondents were also concerned with the currently available diagnostic tools, and problems of misidentification of *B. pseudomallei* as other *Burkholderia* or *Pseudomonas* species. In terms of treatment, the greatest challenge was ensuring adherence and the management of adverse reactions secondary to prolonged courses of antibiotics.

## 5. Challenges in the Control and Management of Melioidosis

It is uncertain whether melioidosis is being under-diagnosed in Hong Kong. A recent study estimated that Hong Kong would expect to see 67 new cases of melioidosis annually [4]. This estimation was much higher than the number of cases observed in our review for the last two decades. As reflected in our local survey, under-diagnosis could be due to the low level of clinical suspicion among clinicians. The diagnosis of melioidosis can be difficult in clinical cases, as *B. pseudomallei* might not be readily isolated from clinical specimens [39], or misidentified as other *Burkholderia* species by certain automated systems [40]. Cases diagnosed to be pulmonary tuberculosis or other bacterial cases of pneumonia in Hong Kong have also been suspected to be melioidosis [6,10,15]. Moreover, we have only included culture-confirmed cases in our review; culture-negative cases confirmed only by serological evidence of infection were not included in the analysis. Since we have observed an increasing number of cases over the last 20 years, continual surveillance of this disease in the territory should be implemented.

Improvements in the laboratory diagnosis of melioidosis are urgently needed. Molecular techniques, involving 16S rRNA or *groEL* gene sequencing, would be useful for identification, in particular, to differentiate *B. pseudomallei* from other closely related *Burkholderia* species, including *B. thailandensis* [24,41]. As observed in our survey, MALDI-TOF MS is being increasingly used in Hong Kong microbiology laboratories. This technique has recently been shown to be an accurate method for the identification of *B. pseudomallei* [42]. Preliminary data also showed that profiling metabolites from various lipid classes is a potential biomarker for the diagnosis of melioidosis [39].

The case fatality rate observed in Hong Kong was higher than that observed in other high-income countries. As indicated by the results of our survey, the relatively low number of cases detected in Hong Kong led to a low level of clinical suspicion and possibly a delay in diagnosis. Empirical treatment of melioidosis, for example, is seldom initiated for patients with community-acquired pneumonia in Hong Kong. Awareness of this disease should be raised, and the diagnosis should be particularly considered in patients with compatible clinical manifestations and associated epidemiological risk factors.

The growing number of cases of melioidosis could partially be explained by the aging population and the increase in immunocompromising diseases and therapies. Currently, there is no local guideline in the prevention and management of the disease in patients at high risk of complications and mortality from melioidosis. Recommendations have been issued for the workup of melioidosis in seropositive patients initiating immunosuppressive therapy and symptomatic seropositive immunocompromised patients [36]. Routine serological testing of antibodies against *B. pseudomallei* is currently not performed in immunocompromised patients in Hong Kong. The only published local seroprevalence surveys were done in the 1980s. A study should, therefore, be considered to evaluate the current seroprevalence rate of melioidosis in Hong Kong, and to determine whether routine screening is necessary for immunocompromised patients in Hong Kong.

## 6. Conclusions

Melioidosis is endemic in the urban city of Hong Kong. Although the observed incidence was lower than in other neighboring countries and regions, there was an increasing number of cases in the last 20 years. Surveillance of the disease should be considered, and more effort should be in place to raise clinicians’ awareness of the disease.

## Figures and Tables

**Table 1:** Published reports of culture-confirmed human cases of melioidosis.

Author	Gender/Age	Underlying Diseases	Site of Involvement	Treatment	Outcome
Sridhar 2015 [21]	Male/55	Acute myeloid leukemia with prolonged neutropenia	Bacteremia and pulmonary	Ceftazidime	Died
Sridhar 2015 [21]	Male/57	Follicular lymphoma with prolonged neutropenia	Bacteremia, bursitis and pulmonary	Meropenem, followed by ceftazidime	Died
Li 2015 [22]	Male/82	Coronary artery disease	Mycotic aneurysm and pneumonia	Ceftazidime for 3 weeks, followed by amoxicillin-clavulanate and doxycycline for 3 months	Survived
Lee 2013 [23]	Male/40	X-linked chronic granulomatous disease and concomitant miliary tuberculosis	Not available	Not available	Survived
Woo 2003 [24]	Female/84	Diabetes and bronchiectasis	Bacteremia and pulmonary	Ceftazidime for 2 weeks, followed by amoxicillin-clavulanate for 20 weeks	Survived
Tsang 2001 [25]	Male/51 (Nepalese)	Diabetes	Empyema thoracis	Imipenem for 2 weeks, followed by ciprofloxacin for 7 months, then switched to amoxicillin-clavulanate due to resistance to ciprofloxacin	Survived
Que 1991 [26]	Male/53	Nil	Abscesses in prostate, kidneys, liver, spleen and lungs	Not available	Died
Woo 1987 [27]	Male	Nil	Bacteremia and prostatic abscess	Trimethoprim-sulfamethoxazole	Survived
So 1984 [10]	Female/77	Diabetes	Bacteremia and splenic abscess	Penicillin, gentamicin and metronidazole	Died
So 1984 [10]	Male/62	Diabetes	Bacteremia	Chloramphenicol and tetracycline	Survived
So 1984 [10]	Male/72	Diabetes	Bacteremia and pulmonary	Amikacin	Died
So 1984 [10]	Female/81	Diabetes	Bacteremia and pulmonary	Ampicillin	Died
So 1984 [10]	Male/32	On steroid for pemphigus vulgaris	Bacteremia and pulmonary	Anti-tuberculous drugs	Died
So 1983 [14]	Female/32	Systemic lupus erythematosus, on steroid and azathioprine	Pulmonary	Ceftazidime for 2 months	Survived
